# TRESK background potassium channel is not gated at the helix bundle crossing near the cytoplasmic end of the pore

**DOI:** 10.1371/journal.pone.0197622

**Published:** 2018-05-15

**Authors:** Miklós Lengyel, Gábor Czirják, Péter Enyedi

**Affiliations:** Department of Physiology, Semmelweis University, Budapest, Hungary; Tel Aviv University Sackler Faculty of Medicine, ISRAEL

## Abstract

Two-pore domain K^+^ channels (K_2P_) are responsible for background K^+^ currents and regulate the resting membrane potential and cellular excitability. Their activity is controlled by a large variety of physicochemical factors and intracellular signaling pathways. The majority of these effects converge on the intracellular C-terminus of the channels, resulting in the modification of the gating at the selectivity filter. Another gating mechanism, the activation gate at the helix bundle crossing is also well documented in other K^+^ channel families, however, it remains uncertain whether this type of gating is functional in K_2P_ channels. The regulation of TWIK-related spinal cord K^+^ channel (TRESK) is different from the other K_2P_ channels. Regulatory factors acting via the C-terminus are not known, instead channel activity is modified by the phosphorylation/dephosphorylation of the unusually long intracellular loop between the 2^nd^ and 3^rd^ transmembrane segments. These unique structural elements of the regulation lead us to examine channel gating at the bundle crossing region. Ba^2+^ was applied to the intracellular side of excised membrane patches and the characteristics of the channel block were determined. We compared the kinetics of the development of Ba^2+^ block when the channels were phosphorylated (inhibited) or dephosphorylated (activated) and also in different mutants mimicking the two functional states. Neither the phosphorylation/dephosphorylation nor the point mutations influenced the development of Ba^2+^ block, suggesting that the conformational changes of the bundle crossing region do not contribute to the phosphorylation-dependent gating of TRESK.

## Introduction

Two-pore domain K^+^ channels (K_2P_) are the molecular correlates of background potassium currents. These channels are responsible for the resting membrane potential and play a role in the regulation of cellular excitability in many cell types. To date, 15 mammalian K_2P_ subunits have been identified. These channels are regulated by a variety of physico-chemical factors and signaling pathways (for detailed reviews see [1, 2]).

TWIK-Related spinal cord K^+^ channel (TRESK, K_2P_18.1), was originally cloned from human spinal cord [3]. TRESK expression is most abundant in the primary sensory neurons of the dorsal root and trigeminal ganglia [4–6]. Elevation of the cytoplasmic Ca^2+^ concentration activates TRESK. The calcium ion does not act on TRESK via the direct binding to the channel protein, but the calcium/calmodulin-dependent phosphatase calcineurin activates the K^+^ current by dephosphorylating S264 and the S276 serine cluster [7]. These residues are constitutively phosphorylated under basal conditions by protein kinase A and microtubule-affinity regulating kinases (MARK), resulting in channel inhibition [8, 9].

In the case of voltage-gated (K_v_) and inwardly-rectifying (K_ir_) K^+^ channels it is widely accepted that transition between the non-conducting to the conducting states is mediated by three distinct mechanisms (for review, see [10] and [11]). Most of our knowledge regarding the gating of K^+^ channels derives from experiments using K_v_ channels as models. Much less is known about the processes responsible for the gating of channels in the K_2P_ family (for a recent review see [12]). In an early study using the *Drosophila* K_2P_ channel KCNK0 as a model, it was demonstrated that regulation of this channel by protein kinases involves conformational changes in the selectivity filter similar to the C-type inactivation described in K_v_ channels [13]. Numerous studies have demonstrated that the gating of various K_2P_ channels by a variety of other stimuli (such as changes in the intra- or extracellular pH, temperature or membrane tension) involves a similar process affecting the selectivity filter [14–18].

The presence of a helix bundle crossing gate in K_2P_ channels was first hypothesized to explain the voltage-dependent gating of TASK-3 [19] (for a recent and detailed investigation of voltage-dependent gating of K_2P_ channels, see [20]). The existence of a functional activation gate in K_2P_ channels was also hypothesized on the basis of a study using a chimeric channel constructed from the core of KCNK0 and the voltage-sensing domain of the *Shaker* K_v_ channel [21]. However, functional studies performed on TREK-1 indicated that the bundle-crossing gate is permanently open [17, 18]. High resolution crystal structures of TREK-1 and TREK-2 have confirmed the results of these functional studies [22, 23]. Extrapolating these results led to the currently accepted general view that the gating of K_2P_ channels is confined to the selectivity filter.

TRESK is a unique member of the K_2P_ channel family, with a remarkably low amino acid sequence identity (19%) compared to the other K_2P_ channels. The channel has an unusually large intracellular loop and a short C-terminus compared to other members of the K_2P_ family. Known physiological stimuli regulating the activity of the channel by phosphorylation or dephosphorylation converge on this uniquely large intracellular loop of the channel. To date, no mechanism influencing TRESK activity via the C-terminus has been identified. Furthermore, the single channel properties of TRESK are also exceptional; in symmetric 140 mM K^+^ at depolarized membrane potentials TRESK activity is characterized by square wave like openings, but when the membrane potential was hyperpolarized the channel produced bursts of very short openings (mean open time shorter than 0.5 ms) [5, 7]. Based on these unique structural features, regulatory properties and asymmetrical single channel behavior, we decided to investigate whether the phosphorylation of TRESK changes the channel activity by the constriction of the ion conducting pathway near the intracellular entrance of the channel pore.

The existence of the activation gate was first hypothesized on the basis of experiments where barium ions were applied intracellularly to squid axon and the ion was able to block voltage-gated K^+^ currents (via binding to the selectivity filter) only after opening of the channels by depolarization [24]. The site of barium binding has been identified using crystal structures of the bacterial K^+^ channel KCsA as the innermost K^+^ binding site of the selectivity filter [25, 26]. This site is composed of 4 main chain carbonyl oxygen atoms and by the hydroxyl oxygen groups of 4 threonine amino acid residues. These threonine residues are conserved in the pore domain consensus sequence (**T**V(I)GY(F)G) of most potassium channels (including TREK-1 and TRESK). Mutating these threonines to serine in TREK-1 lead to channels resistant to block by barium [27]. Application of intracellular Ba^2+^ has been used to examine the role of the bundle crossing in both K_ir_ [28] and K_2P_ [21] channels. We have used a similar approach to examine the gating properties of TRESK. Our results indicate that the phosphorylation of TRESK decreases channel activity by a mechanism different from the variation of pore diameter at the bundle crossing gate.

## Materials and methods

### Chemicals and reagents

Chemicals of analytical grade were purchased from Sigma (St. Louis, MO, USA), Fluka (Milwaukee, WI, USA) or Merck (Whitehouse Station, NJ, USA). Enzymes and kits for molecular biology applications were purchased from Thermo Scientific (Waltham, MA, USA), New England Biolabs (Beverly, MA, USA) and Stratagene (La Jolla, CA, USA). Ionomycin (calcium salt) was purchased from Enzo Life Sciences (Farmington, NY, USA), dissolved in DMSO as a 5 mM stock solution and stored at -20 ^o^C. Protein kinase A (from bovine heart) was purchased from Sigma, dissolved in distilled water and stored in aliquots at -20 ^o^C.

### Plasmids

The cloning of mouse TRESK and the generation of the different mutant channels used in this study (mouse TRESK S276A, S264E, S276E and S264,276E) have been described previously [7, 9].

The T127S mutant of mouse TRESK was created with the QuikChange site-directed mutagenesis kit (Stratagene, La Jolla, CA, USA) according to the manufacturer’s instructions. The plasmid coding mouse TREK-1 channel was kindly provided by Professor M. Lazdunski and Dr. F. Lesage. The subcloning of the coding sequence of TREK-1 into pcDNA3.1 expression vector was described previously [29]. For expression in mammalian cells, wild type and mutant TRESK channels were subcloned into the pIRES-CD8 vector. The plasmid coding human K_v_1.3 channel was a generous gift from Professor Gy. Panyi. For expression in *Xenopus laevis* oocytes, plasmids were linearized and used for *in vitro* cRNA synthesis using the Ambion mMESSAGE mMACHINE™ T7 *in vitro* transcription kit (Ambion, Austin, TX). The structural integrity of the RNA was checked on denaturing agarose gels. The constitutively active MARK2 mutant tagged with Glutathione S-transferase (GST) was described previously [8].

### Cell culture, transient transfection

Cell culture dishes were purchased from Greiner Bio-One GmbH (Kremsmuenster, Austria). HEK293T cells were obtained from ATCC (Manassas, VA. Catalogue number: CRL-3216). Cells were seeded at a density of 20.000–100.000 cells per 35 mm dish 48h prior to transfection in 10% bovine serum in Dulbecco’s modified Eagle’s medium (DMEM). Cells were transfected using Lipofectamine2000 transfection reagent (Invitrogen, Carlsbad, CA, USA) and UltraMEM Reduced Serum Medium according to the manufacturer’s instructions. DMEM, UltraMEM and fetal bovine serum were purchased from Lonza (Basel, Switzerland). Cells were transfected with 1–2.5 μg DNA (depending on channel type) per 35 mm dish and used for experiments 24–48 hours after transfection. The plasmids encoding mouse TREK-1 and human K_v_1.3 were cotransfected with a plasmid coding CD8. Transfected cells were identified using anti-CD8 Dynabeads (Thermo Fisher Scientific).

### Production and purification of recombinant MARK2

The constitutively active GST-MARK2 fusion construct was expressed in the BL21 strain of *E*. *coli*. Solution A contained the following: 50 mM Tris-HCl (pH 7.5), 200 mM NaCl, 1 mM β-mercaptoethanol, 1 mM PMSF and 2 mM benzamidine. Bacteria were sonicated in solution A supplemented with 5 mM CHAPS. After lysis of the bacteria, the fusion protein was affinity-purified with glutathione-agarose resin (Sigma). GST-MARK2 was eluted from the resin with solution A containing 20 mM reduced glutathione. The purified kinase was then dialysed against solution A containing 50% glycerol and stored at -20 ^o^C until use.

### Preparation and microinjection of *Xenopus* oocytes

*Xenopus laevis* oocytes were prepared as previously described [30]. For expression of the different channels oocytes were injected with 50 nl of cRNA one day after defolliculation. Injection was performed with the Nanoliter Injector (World Precision Instruments, Saratosa, FL). *Xenopus laevis* frogs were housed in 50 L tanks with continuous filtering and water circulation. The room temperature was 19°C. Frogs were anesthetized with 0.1% tricaine solution and killed by decerebration and pithing. 2 frogs were used for the experiments. All experimental procedures involving animals were conducted in accordance with state laws and institutional regulations. All experiments were approved by the Animal Care and Ethics Committee of Semmelweis University (approval ID: XIV-I-001/2154-4/2012).

### Two-electrode voltage clamp experiments

Two-electrode voltage clamp experiments were performed 2–3 days after the microinjection of cRNA into *Xenopus* oocytes, as previously described [7]. The holding potential was set to 0 mV. Background potassium currents were measured at the end of 300 ms long voltage steps to −100 mV applied every 4 s. The low potassium recording solution contained (in mM): NaCl 95.4, KCl 2, CaCl_2_ 1.8, HEPES 5 (pH 7.5, adjusted by NaOH). The high potassium solution contained 80 mM K^+^ (78 mM NaCl of the low potassium solution was replaced with KCl). Solutions were applied to the oocytes using a gravity-driven perfusion system. Experiments were performed at room temperature (21 ^o^C). Data were analyzed by pCLAMP 10 software (Molecular Devices, Sunnyvale, CA, USA).

### Patch clamp recording

Pipettes were pulled from thick-walled borosilicate glass (Standard Glass Capillaries, 4 in., 1.2 / 0.68 OD/ID, Filament/Fire Polished (Item number: 1B120F-4) from World Precision Instruments, Sarasota, Florida) by a P-87 puller (Sutter Instrument Co., Novato, CA, USA) and fire polished. Pipettes were filled with pipette solution and connected to the headstage of an Axopatch-1D patch clamp amplifier (Axon Instruments, Inc., Foster City, CA, USA). Experiments were carried out at room temperature (21°C). Solutions were applied using a gravity-driven perfusion system. Data were digitally sampled by Digidata 1200 or a DigiData 1550B (Axon Instruments, Inc.). Data were analyzed by pCLAMP 10 software. Cut-off frequency of the eight-pole Bessel filter was adjusted to 200 Hz and data were acquired at 2 kHz. TREK-1 and TRESK currents were recorded in the gap-free mode at +60 mV. K_v_1.3 current was measured by stepping the membrane potential to +40 mV for 250 ms from a holding potential of -80 mV.

Currents were recorded from excised patches in the inside-out configuration. The pipette solution contained (in mM): 136 NaCl, 4 KCl, 5 EGTA, 1 MgCl_2_ and 10 HEPES (pH 7.4 adjusted with NaOH). Pipette resistances were 3–7 MΩ when filled with pipette solution. Bath solution contained (in mM): 140 KCl, 1 MgCl_2_ and 10 HEPES (pH 7.1 adjusted with KOH). In the case of the bath solution containing Ba^2+^, 1 mM BaCl_2_ was added to the solution before the adjustment of the pH of the solution. The determination of the size of the K^+^ current was done by transiently substituting the bath solution with a K^+^-free bath solution (140 NaCl, 1 MgCl_2_ and 10 HEPES, pH 7.1 adjusted with NaOH) and subtracting the amplitude of the measured current in the K^+^-free bath solution from the current measured in high K^+^.

### Statistics and calculations

Results are expressed as means±S.E.M. The number of patches measured in each group is given in the text and also on the figures. Time constants for the inhibition of the K^+^ current by Ba^2+^ was determined by fitting the data with a double-exponential equation (as in previous studies examining internal barium block of K^+^ channels [21, 28]). Statistical significance was determined using the Mann-Whitney U test or the Student’s *t* test (whichever was appropriate) for independent samples. Multiple groups were compared using either the Kruskal-Wallis test followed by multiple comparisons of mean ranks for all groups or ANOVA followed by Tukey’s *post hoc* test. Results were considered to be statistically significant at p<0.05. Statistical calculations were done using Statistica (version 13.2, Dell Inc., Tulsa, OK, USA).

## Results

### Blocking of K_v_1.3 channels by intracellular Ba^2+^ depends on the conformation of the bundle crossing gate. Closing of the gate results in the trapping of Ba^2+^ ion

**Fig 1:**
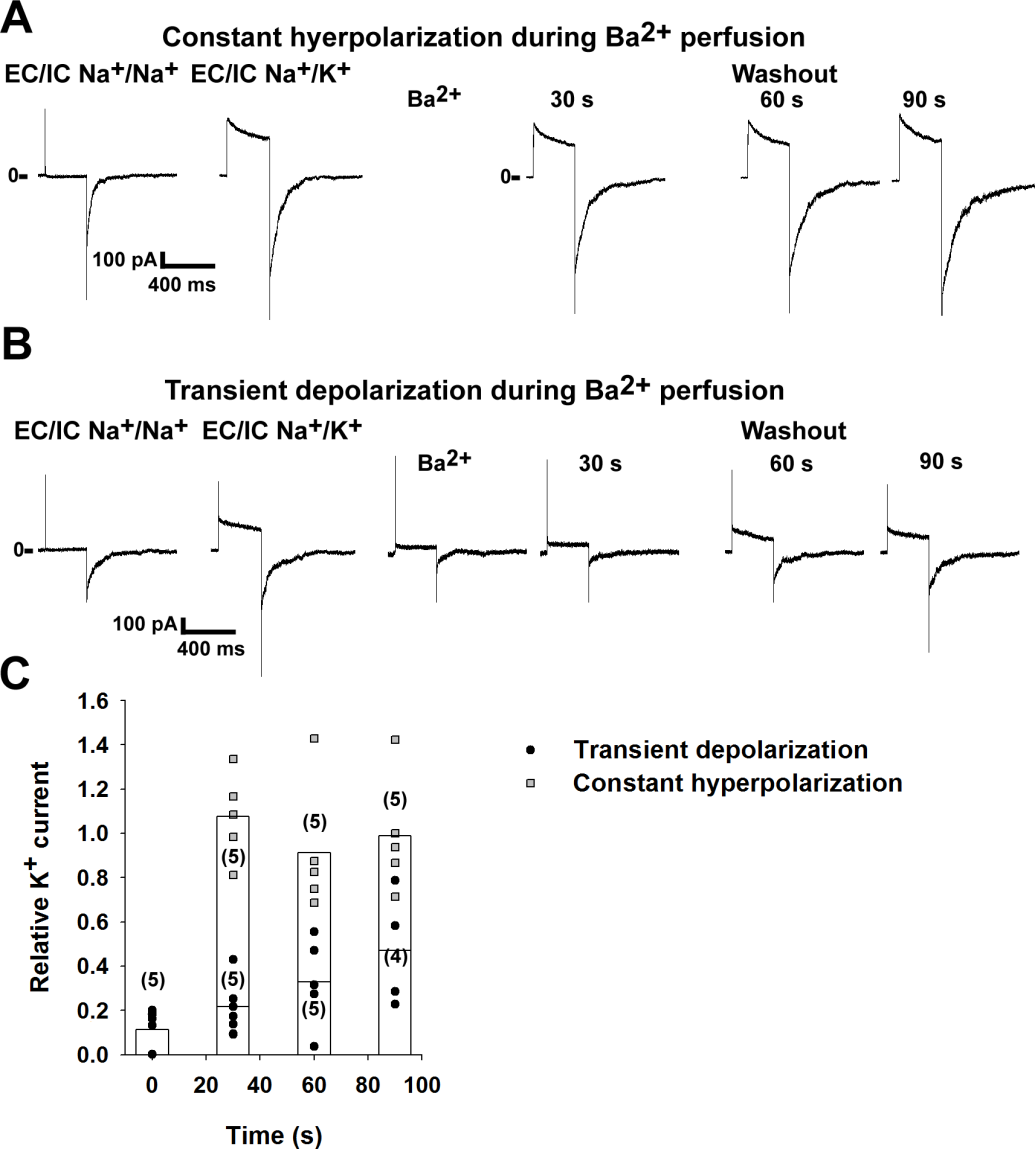
Barium ions can be trapped in the K_v_1.3 channel pore. K_v_1.3 channels were expressed in HEK293T cells. The currents were recorded in inside-out excised patches. Voltage-dependent currents were measured by depolarizing the membrane to +40 mV for 250 ms. The holding potential was -80 mV. Currents were measured at the end of the +40 mV voltage step. K_v_1.3 current was calculated by subtracting the current measured in K^+^-free bath solution from the value measured in the high K^+^ solution. The currents after the administration of Ba^2+^ were normalized to the value measured before the application of the blocker (on panel C). A, Representative recording showing the application of Ba^2+^ (1 mM) to the intracellular surface of the patches while keeping the holding potential at -80 mV (*constant hyperpolarization*). Afterwards the blocker was removed from the bath solution and the current was measured every 30 s by depolarizing steps to +40 mV. B, Representative recording showing the effect of Ba^2+^ (1 mM) applied to the intracellular surface of inside-out patches when the membrane was depolarized to +40 mV (*transient depolarization*). Barium was then removed from the bath solution while the holding potential was -80 mV. Voltage-gated K^+^ currents were measured every 30 s, as in panel A. C, The normalized currents after the application of Ba^2+^ are plotted as a function of time. The channels were opened by transient depolarization to +40 mV (black circles) or closed at constant -80 mV (grey squares) in the presence of Ba^2+^. Data are plotted as scatter plot, the averages of the different groups are plotted as columns. The differences between the groups were significant at all examined time points (p<0.05, statistical analysis was performed using Kruskal-Wallis ANOVA followed by pairwise comparison using the Mann-Whitney U test).

It has been previously reported that barium ions block the pore of potassium channels by binding to the selectivity filter [24, 28]. If Ba^2+^ is applied to the intracellular side of K^+^ channels, it can only reach the selectivity filter by passing through the activation gate, formed by helices at the bundle crossing region. In the case of the voltage-gated K^+^ channels, it is well documented that gating of the channel involves movement of the bundle crossing. To confirm that the Ba^2+^ block method is suitable for the evaluation of the activation gate, we have expressed human K_v_1.3 channels in HEK293T cells and examined the block of the channel by Ba^2+^ in excised inside-out patches. After measuring the K_v_1.3 current in the patch (at +40 mV), we applied 1 mM Ba^2+^ to the bath solution while holding the membrane potential at -80 mV. If the Ba^2+^ was washed out from the bath solution while keeping the membrane hyperpolarized, i.e. without opening the channels, the amplitude of the current to a subsequent test depolarizing step was similar to the current measured before application of Ba^2+^ (n = 5 patches, for representative recording see Fig 1A. For a summary, see the grey squares on Fig 1C). This indicates that Ba^2+^ did not reach its site of action, the selectivity filter, while the inner gate was closed. However, when the channels were opened by depolarization in the presence of Ba^2+^, the K^+^ current was blocked (88.8±3.4% inhibition, n = 5 patches. For representative recording, see Fig 1B). After determining the degree of the block, Ba^2+^-free solution was applied while keeping the channels closed by holding the membrane potential at -80 mV. The size of the K^+^ current was determined every 30 s during the washout (Fig 1B). The current remained partially blocked (n = 4–5 patches for each time point, see the black circles on Fig 1C for a summary). The current of the partially blocked channels was significantly smaller than the current of the unblocked channels at all examined time points (p = 0.012 for the 30 and 60 s time point, p = 0.037 for the 90 s time point. Statistical analysis was performed using Mann-Whitney U test). Accordingly, we have reproduced the previous results in our experimental system that changes in the conformation of the activation gate determine the kinetics of the recovery from the block induced by the intracellularly applied Ba^2+^. Therefore, Ba^2+^ is an appropriate tool to examine the role of the activation gate in K^+^ channels.

### The rate of Ba^2+^ inhibition does not depend on the functional state of TREK-1

**Fig 2:**
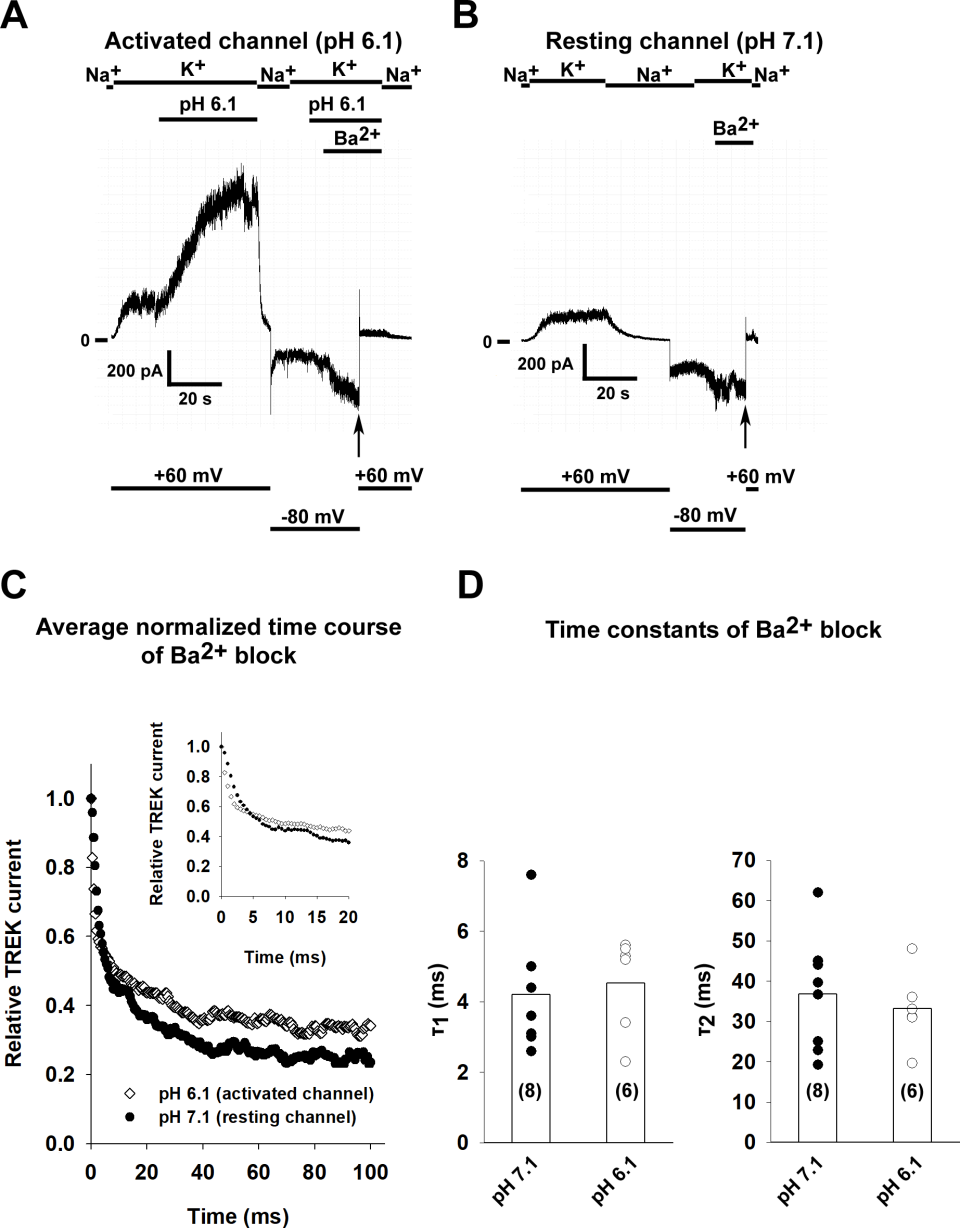
The kinetics of Ba^2+^ block is not affected by the activation of TREK-1 channel. A, TREK-1 channels were expressed in HEK293T cells and recordings were done on excised inside-out patches. TREK-1 currents were measured at +60 mV by switching the K^+^-free bath solution to a high K^+^ solution (solution changes are marked with bars above the graphs). In this representative recording, TREK-1 channels were activated by perfusing the intracellular side of the patch with acidic solution (pH 6.1) as shown on the graph. Barium (1 mM) was applied at a holding potential of -80 mV and block was initiated by depolarizing the membrane to +60 mV (application of Ba^2+^ is marked by a bar above the recording and changes in the membrane potential are shown under the recording). B, The pH of the bath solution was 7.1 throughout the experiment. In this representative recording, Ba^2+^ (1 mM) was applied at a holding potential of -80 mV and block was initiated by depolarizing the membrane to +60 mV (see the *vertical arrow*, application of Ba^2+^ is marked by a bar above the recording and changes in the membrane potential are shown under the recording.). C, The kinetics of Ba^2+^ induced TREK-1 block were determined for both the resting (perfused with pH 7.1 solution) and activated (pH 6.1) channel (n = 8 and 6 patches). The averaged normalized curves are plotted for both groups. The inset shows the onset of Ba^2+^ block at an early time period with higher temporal resolution. D, The current recordings of the Ba^2+^ block recorded at different pH values were fitted with a double exponential equation. The time constants of the fitted equations are plotted as a scatter plot. The average values are plotted as columns. Differences between the groups were not statistically significant.

Kinetic analysis of block by intracellularly applied tetrapentylammonium ions revealed that the bundle crossing in TREK-1 stays open even when the channel is in the nonconductive state [18]. This model was subsequently confirmed by the comparison of high resolution crystal structures of the channel in different conformations [23]. Considering that the same threonine amino acid residues are necessary for the tetrapentylammonium block of TREK-1 as for the inhibition of the channel by barium, the rate of inhibition by Ba^2+^ should be the same for the resting and activated state. TREK-1 currents were measured at +60 mV by perfusing the patches with high K^+^ bath solution. In accordance with the previous results, acidification of the solution at the intracellular side of the membrane activated TREK-1 current (see Fig 2A for a representative recording). Barium ions (1 mM) were applied to the perfusing solution while the patch was held at -80 mV to prevent the Ba^2+^ from reaching its site of action, the selectivity filter. Blockage of the channels by Ba^2+^ was initiated by depolarizing the patch to +60 mV (a similar experimental protocol was previously used to examine the function of the bundle crossing in K_ATP_ and other K_2P_ channels [21, 28]). The Ba^2+^ block was measured in solutions with a pH of 7.1 (for a representative recording, see Fig 2B) or 6.1 (n = 8 and 6 patches). The averaged normalized curves of the Ba^2+^ block can be seen in Fig 2C (the inset shows the early time period of inhibition at a higher temporal resolution). Time constants for the Ba^2+^ block were determined by fitting the data with a double-exponential equation. The time constants for the capacitative transient caused by depolarization to +60 mV in the absence of Ba^2+^ were 2 orders of magnitude smaller than the time constants measured in the presence of Ba^2+^. As expected for a channel with an open bundle crossing, there was no difference (p = 0.69 and p = 0.59 for τ_1_ and τ_2_ respectively, Student’s t test) in the time constants of the Ba^2+^ block (for a summary, see Fig 2D).

### Constitutively active MARK2 kinase and protein kinase A (PKA) inhibits TRESK current in excised patches

**Fig 3:**
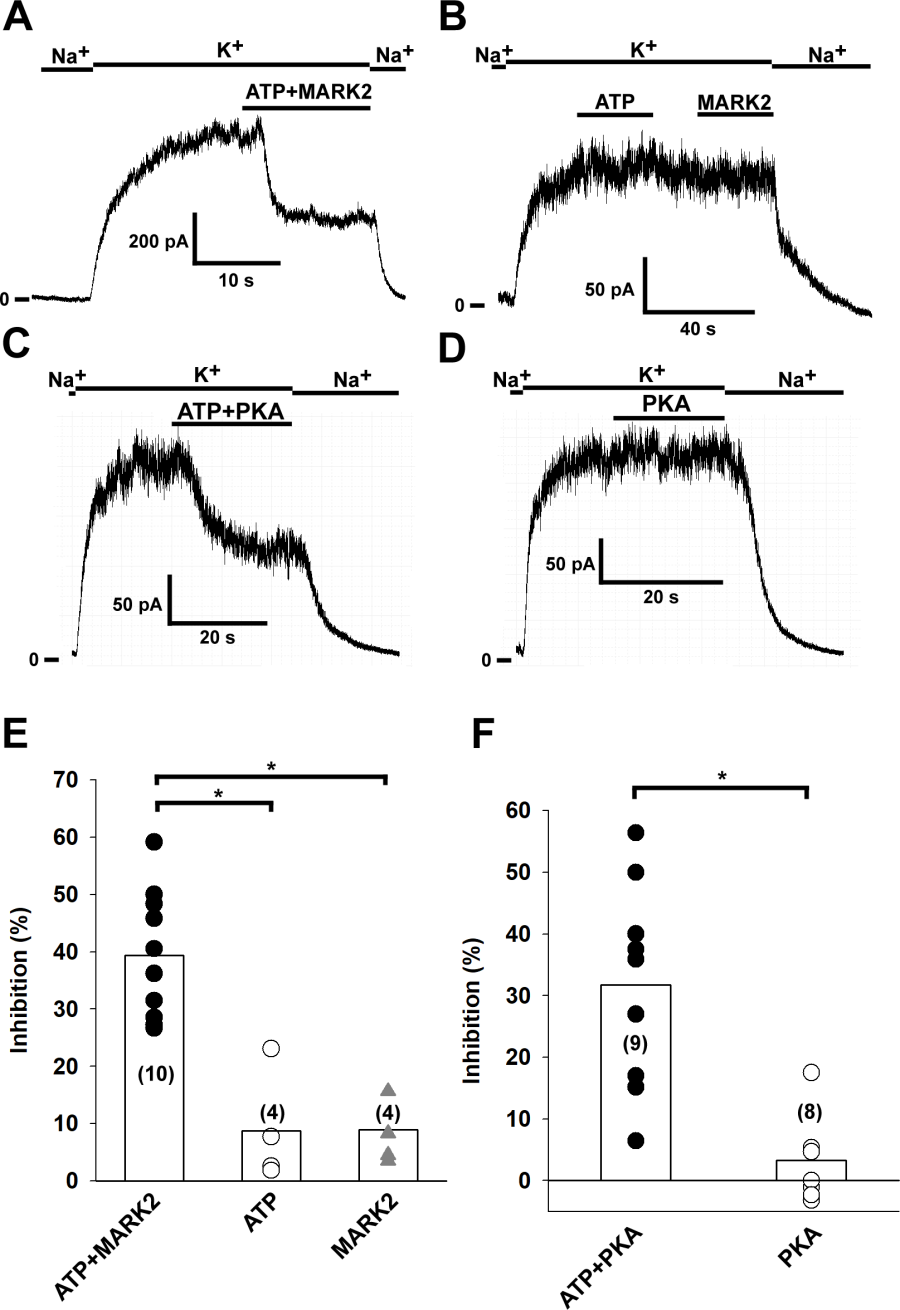
TRESK channel is inhibited by MARK2 and PKA phosphorylation. Mouse TRESK channels were expressed in HEK293T cells. Experiments were done on excised inside-out patches. TRESK currents were measured at +60 mV by switching the K^+^-free bath solution to a high K^+^ solution (solution changes are marked with bars above the graphs). Application of ATP and purified MARK2 (16 μg/ml) or separate application of ATP or MARK2 are marked by the bars above the recordings. A, Representative recording showing that application of MARK2 and 2 mM ATP leads to inhibition of TRESK current by phosphorylation. B, Representative recording showing that separate application of 2 mM ATP and MARK2 does not have an effect on TRESK current. C, Representative recording showing that application of PKA (30 U/ml) in the presence of 1 mM cAMP, 1 mM DTT and 2 mM ATP inhibits TRESK current by phosphorylating the channel. D, Representative recording showing that application of PKA (30 U/ml) in the presence of 1 mM cAMP, 1 mM DTT, but in the absence of ATP has no effect on TRESK current. E, The effects of ATP, MARK2 and ATP+MARK2 on TRESK current have been summarized as a scatter plot. The averages of each group are plotted as column graphs. Statistical significant differences between the groups (p<0.05, determined by Kruskal-Wallis test followed by multiple comparisons of mean ranks) are marked with asterisks. F, The effects of PKA and PKA+ATP on TRESK current have been summarized as a scatter plot. The averages of each group are plotted as column graphs. Statistical significant differences between the groups (p<0.05, determined by the Mann-Whitney U test) are marked with asterisks.

We have previously reported that MARK2 and PKA inhibit the whole cell TRESK current in *Xenopus* oocytes via phosporylation of serine 276 (by MARK2) and serine 264 (by PKA) residues located in the intracellular loop of the channel [8, 9]. In this study, we have examined the effect of recombinant constitutively active MARK2 kinase and PKA on TRESK current in inside-out patches. When purified MARK2 kinase (16 μg/ml kinase dissolved in high K^+^ bath solution) and 2 mM ATP were applied to the patch, TRESK current was inhibited by 39.4±3.4% (n = 10 patches, for representative recording see Fig 3A). This inhibition implies that MARK2 was able to phosphorylate the channels found in the excised patch. To demonstrate that the inhibition was a consequence of the phosphorylation of TRESK, we applied 2 mM ATP or MARK2 separately (n = 4 patches, for representative recording see Fig 3B). The amplitude of TRESK current was not affected in either case. The results obtained with MARK2 are summarized in Fig 3E, the inhibition by the combined application of MARK2 and ATP was significant compared to the controls (p = 0.008 compared to application of ATP alone, and p = 0.02 compared to application of MARK2 without ATP, Kruskal-Wallis test followed by multiple comparisons of mean ranks for all groups). We have also examined the effect of PKA on TRESK current. When PKA holoenzyme (30 U/ml bath solution; with 1 mM cAMP and 1 mM DTT added to activate the kinase) was applied to the patches in the presence of 2 mM ATP, TRESK current was inhibited by 31.7±5.2% (n = 9 patches, for representative recording see Fig 3C). To show that the inhibition was the consequence of phosphorylation by PKA, we applied the same bath solution to excised patches, but in the absence of ATP. As expected, no inhibition of TRESK current was observed (3.3±2.2% inhibition, n = 8 patches). The results obtained with PKA are summarized in Fig 3F. The inhibition observed after combined application of PKA and ATP was significant compared to the control (p = 0.0018, Mann-Whitney U test).

### Phosphorylation of TRESK does not change the rate of Ba^2+^ inhibition

Next we asked whether phosphorylation of the intracellular loop by MARK2 kinase and PKA has an effect on the conformation of the bundle crossing gate by measuring the rate of Ba^2+^ block in inside-out excised patches. To verify that the inhibitory barium binding site in TRESK is located near to the selectivity filter similarly to the site described in TREK-1, we mutated the threonine 127 residue in the first pore domain of TRESK to serine (mutation of the corresponding residue in TREK-1 lead to the loss of barium block [27]). Wild type and T127S mutant TRESK channels were expressed in *Xenopus* oocytes and their sensitivity to Ba^2+^ was determined using the two-electrode voltage clamp method. The current of the wild type channel was efficiently inhibited by Ba^2+^ (1 mM, 64.4±7.3% inhibition, n = 5 oocytes; p = 0.04), while the T127S mutant was resistant to application of barium (7.2±3.0% inhihition, n = 6 oocytes. p = 0.12). The difference in the degree of inhibition was statistically significant (p = 0.008. Statistical analysis was performed using the Mann-Whitney U test).

**Fig 4:**
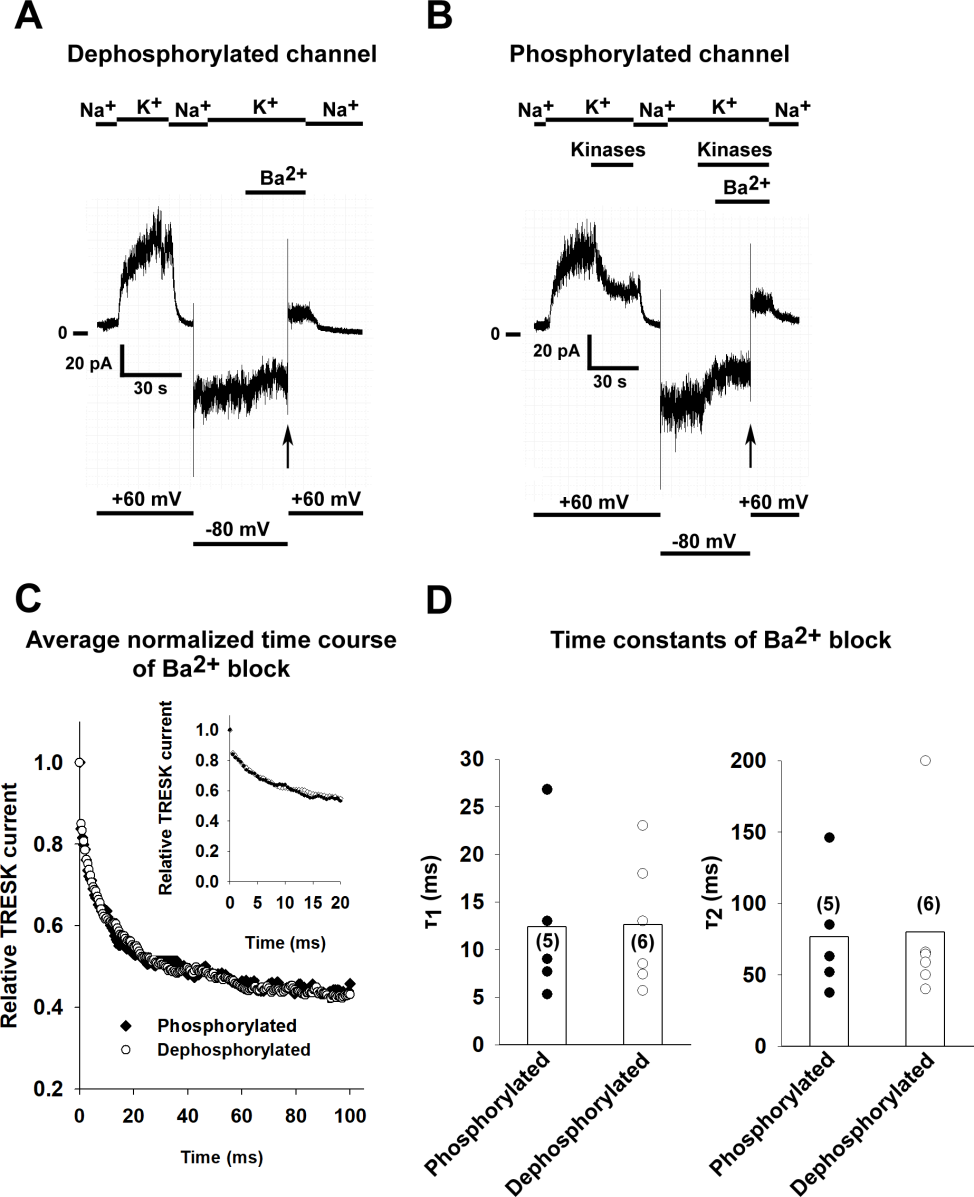
The phosphorylation state of TRESK does not influence the kinetics of Ba^2+^ block. Mouse TRESK channels were expressed in HEK293T cells. Experiments were done on excised inside-out patches. TRESK currents were measured at +60 mV by switching the K^+^-free bath solution to a high K^+^ solution (solution changes are marked with bars above the graphs). Barium (1 mM) was applied at a holding potential of -80 mV and block was initiated by depolarizing the membrane to +60 mV. TRESK channels were either dephosphorylated by application of 0.5 μM ionomycin to the bath solution before recording or phosphorylated by application of purified MARK2 (16 μg/ml), 30 U/ml PKA (1 mM cAMP and 1 mM DTT was added to ensure the enzymatic activity of PKA) and 2 mM ATP before the initiation of the Ba^2+^ block. A, Representative recording, TRESK channels were dephosphorylated before patch excision by application of 0.5 μM ionomycin to the bath solution. Barium (1 mM) was applied at a holding potential of -80 mV and block was initiated by depolarizing the membrane to +60 mV (see the *vertical arrow*, application of Ba^2+^ is marked by a bar above the recording and changes in the membrane potential are shown under the recording). B, Representative recording, TRESK channels were phosphorylated by perfusing the intracellular side of the patch with a bath solution containing both kinases (purified MARK2, PKA, 1 mM cAMP, 1 mM DTT and 2 mM ATP) as shown on the graph. Barium (1 mM) was applied at a holding potential of -80 mV and block was initiated by depolarizing the membrane to +60 mV (see the *vertical arrow*, application of Ba^2+^ is marked by a bar above the recording and changes in the membrane potential are shown under the recording). C, The kinetics of TRESK current inhibition by Ba^2+^ were determined for both the phosphorylated and dephosphorylated channel (n = 6 and 5 patches). The average normalized curves for both groups are plotted. The inset shows the onset of Ba^2+^ with a higher temporal resolution. D, The current recordings of the Ba^2+^ block recorded for both the phosphorylated and dephosphorylated groups were fitted with a double exponential equation. The time constants of the fitted equations are plotted as a scatter plot. The average values are plotted as columns. The difference between the groups was not statistically significant (Student’s t test).

HEK293T cells expressing TRESK channels were treated with 0.5 μM ionomycin, a Ca^2+^ ionophore to the cells before patch excision, which results in the activation of calcineurin and the dephosphorylation of the channel. In order to obtain phosphorylated (inhibited) channels, recombinant MARK2 and PKA (plus 2 mM ATP, 1 mM cAMP and 1 mM DTT) was applied after patch excision, as detailed in Fig 3. The reduction of the current by application of the two kinases was 52.6±2.8% (n = 5 patches). Barium block was initiated by depolarizing the patch from -80 mV to +60 mV (as in the case of TREK-1). For a representative recording of a dephosphorylated and phosphorylated channel, see Fig 4A and 4B respectively. The average normalized curves of the two groups can be seen in Fig 4C. There was no difference in the rate of Ba^2+^ inhibition as summarized in Fig 4D (n = 5 and 6 patches, p = 0.96 and p = 0.92 for τ_1_ and τ_2_ respectively, Student’s t-test), suggesting that phosphorylation of the channel does not restrict the access of Ba^2+^ to the selectivity filter.

### TRESK mutants mimicking different states of the channel have similar kinetics of Ba^2+^ block

**Fig 5:**
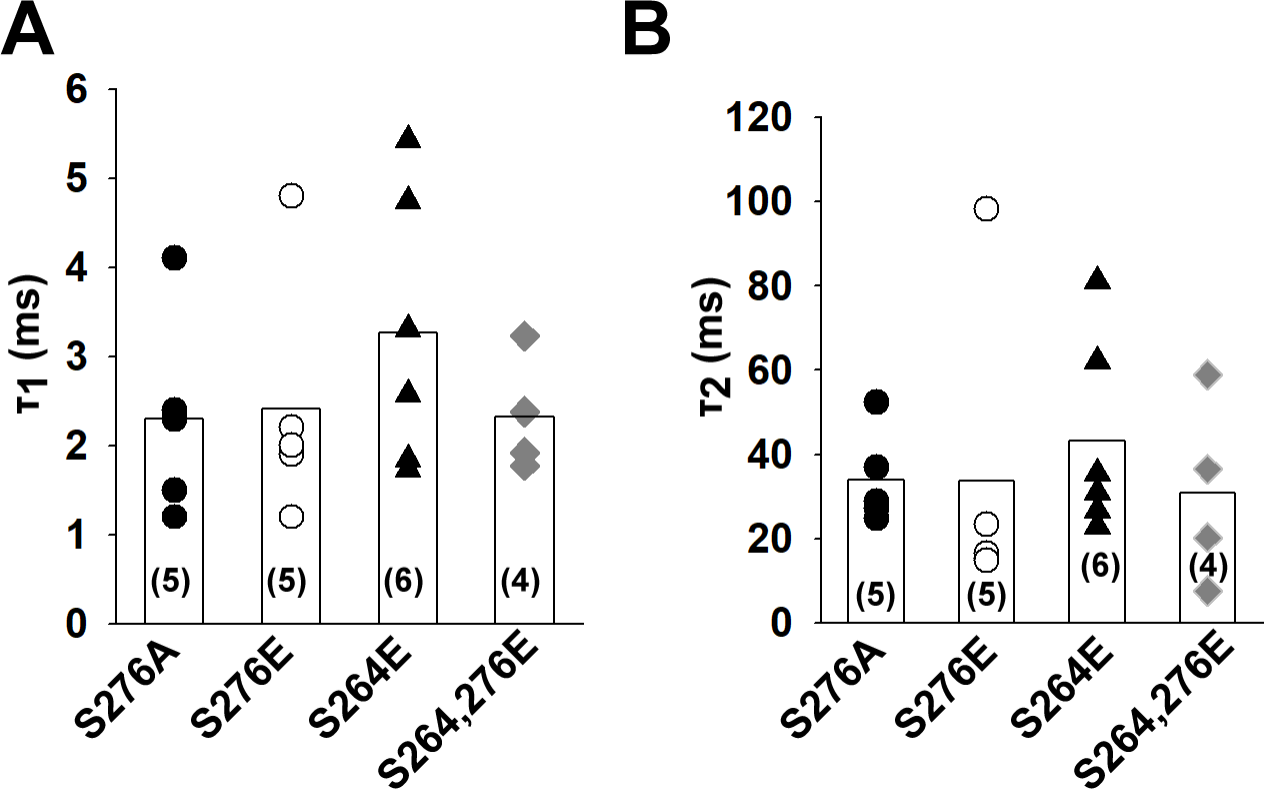
The rate of Ba^2+^ inhibition is similar in TRESK mutants mimicking the phosphorylated and dephosphorylated channel. Mutant mouse TRESK channels were expressed in HEK293T cells. Experiments were done on excised inside-out patches. TRESK currents were measured at +60 mV by switching the K^+^-free bath solution to a high K^+^ solution. Barium (1 mM) was applied at a holding potential of -80 mV and block was initiated by depolarizing the membrane to +60 mV. A and B, The current recordings of the Ba^2+^ block recorded for the different mutants were fitted with a double exponential equation. The time constants of the fitted equations are illustrated as a scatter plot. The average values are plotted as columns. The differences between the groups were not statistically significant (ANOVA).

We have previously reported that mutation of serine 276 residue to alanine in TRESK (mimicking the dephosphorylated state) results in a constitutively active channel [7]. Mutation of serine 264 and serine 276 to glutamate may correspond to the phosphorylated channel. The wild type channel regulated by phosphorylation/dephosphorylation and the appropriate mutants exhibit highly similar pharmacological properties, suggesting that the mutant channels can be used as models of the physiological regulation. To test the possibility that these mutations have an effect on the conformation of the bundle crossing, we have determined the rate of Ba^2+^ block as described in the case of TREK-1 and wild type TRESK (n = 4–6 patches for each mutant). In accordance with the results of the wild type channel, the kinetics of the block was similar (p = 0.51 and p = 0.86 for τ_1_ and τ_2_ respectively, one-way ANOVA) for both the alanine and the glutamate mutants (for a summary, see Fig 5A and 5B). Therefore, the different activity of the mutants under whole cell conditions is not the consequence of the conformation change at the activation gate.

## Discussion

Compared to other K^+^ channels, much less is known about the conformational changes that lead to the opening and closing of K_2P_ channels. Based on the structural and electrophysiological data, it appears that gating of these channels occurs via conformational changes at the selectivity filter. It is an almost general phenomenon that intracellular regulatory stimuli (pH, protein kinases, etc.) converge on the C-terminal intracellular tail of these channels (the regulation of K_2P_ channels is most extensively investigated in the case of the TREK and TASK subfamilies, for review see [1, 2]), which is functionally coupled to the selectivity filter. However, in the case of TRESK, known regulation of channel activity by phosphorylation relies on the intracellular loop between the second and third transmembrane segments (for a review of TRESK regulation, see [31]). Therefore, based on previous results using a chimeric K_2P_ channel where the activation gate was found to be functional [21], we raised the question whether TRESK may differ from the structurally related other K_2P_ channels in a way that the bundle crossing gate plays a role in the gating. To examine the role of the bundle-crossing structure in the gating of different K^+^ channels, we applied Ba^2+^ ions to the intracellular surface of excised inside-out membrane patches. Under these conditions, Ba^2+^ can only reach its site of action, the selectivity filter, when the bundle-crossing gate is open.

To test if Ba^2+^ applied this way was an appropriate tool to examine gating at the bundle-crossing under our experimental conditions, we first examined the characteristics of Ba^2+^ block on a voltage-gated K^+^ channel, K_v_1.3 (the importance of the bundle-crossing gate in K_v_ channels is well documented [32, 33]). As expected (based on previous results), application of the blocker to the intracellular side of the membrane patches only produced an inhibition of the current if the channels were opened by membrane depolarization, confirming that Ba^2+^ is an appropriate tool to examine the activation gate of K^+^ channels.

High resolution crystal structures and electrophysiological experiments using intracellularly applied blockers indicated that the bundle crossing gate is open in TREK-1 even when the channel is closed [18, 23]. Therefore TREK-1 was considered to serve as a negative control for our approach. If the bundle crossing is permanently open, the kinetics of intracellular Ba^2+^ block should be the same regardless of the activity of the channel. To test this hypothesis, we determined the kinetics of Ba^2+^ block on channels in the resting and in the activated state (channels were activated by perfusion of an acidic solution on the intracellular side). The inside-out patches were perfused with Ba^2+^ at a negative membrane potential and the block was rapidly initiated by depolarizing the membrane. As expected on the basis of previous studies, no difference was detected in the kinetics of Ba^2+^ block.

In order to examine the mechanism of TRESK regulation in respect to the gating of the channel, we have to alter channel activity in our experimental system. We have previously reported that MARK2 kinase and PKA phosphorylates TRESK *in vitro* and in *Xenopus* oocytes, leading to inhibition of TRESK activity [8, 9]. In the present study, we tested the effects of purified, constitutively active MARK2 kinase and PKA on TRESK channels in excised inside-out membrane patches. Separate application of MARK2, PKA and ATP had no effect on TRESK current. However, when ATP and one of the kinases were applied simultaneously, a decrease of the TRESK current was observed. Thus in good accordance with our previous *in vitro* and whole cell data, we have demonstrated the inhibitory effect of phosphorylation by both MARK2 and PKA on TRESK activity for the first time under cell-free conditions.

To test our hypothesis that the phosphorylation state of the channel changes the conformation of the bundle crossing gate, we examined the kinetics of Ba^2+^ block on phosphorylated and dephosphorylated channels. The kinetics of Ba^2+^ block were similar in both the phosphorylated and dephosphorylated group. Similar results were obtained with mutant channels mimicking the different phosphorylation states of the channel. These findings are similar to our results obtained with TREK-1, a channel where the helix bundle crossing does not play a role in the gating of the channel. Based on our results, regulation of TRESK by phosphorylation does not affect channel activity by changing the diameter of the ion conduction pathway at the bundle crossing gate. Further studies will be necessary to determine how the phosphorylation state of the intracellular loop of TRESK leads to gating of the channel at the selectivity filter.
